# Role of PH Monitoring in Laryngopharyngeal Reflux Patients with Voice Disorders

**Published:** 2016-11

**Authors:** Swati Maldhure, Ramanathan Chandrasekharan, Amit- Kumar Dutta, Ashok Chacko, Mary Kurien

**Affiliations:** 1 *Department of Otorhinolaryngology, Padhar Hospital, Padhar, Dist Betul, Madhya Pradesh, India.*; 2 *Department of Otorhinolaryngology, Christian Medical College, Vellore, Tamilnadu, India.*; 3 *Department of Gastroenterology, Christian Medical College, Vellore, Tamilnadu, India.*; 4 *Department of Gastroenterology, Madras Medical Mission, Chennai, Tamilnadu, India.*; 5 *Department of Otorhinolaryngology, Pondicherry Institute of Medical Sciences, Puducherry, India.*

**Keywords:** Dysphonia, Esophageal pH Monitoring Received, Gastroesophageal Reflux, Laryngopharyngeal Reflux, date: 28 Feb 2016

## Abstract

**Introduction:**

Laryngopharyngeal reflux (LPR) is considered an important cause of voice disorder. We aimed to determine the frequency of LPR in patients with voice disorder and the association between Koufman Reflux Symptom Index (RSI), Reflux Finding Score (RFS), gastroesophageal reflux disease (GERD), and proximal acid reflux in these patients.

**Materials and Methods:**

We performed a prospective study in patients aged more than 18 years presenting at the ear, nose, and throat (ENT) clinic with a change in voice lasting more than 3 weeks. All patients underwent nasopharyngolaryngoscopy and a dual-probe esophageal pH study. LPR was diagnosed by a Koufman RSI of >13 and/or RFS of >7. GERD was diagnosed according to a DeMeester Johnson score of >14.7. Proximal acid reflux was diagnosed if acid exposure time was >0.02% in a proximal pH probe.

**Results:**

The study included 30 patients with a voice disorder. The mean age of participants was 38.5 years and 40% of patients were female. Using either of the two criteria, LPR was present in 46.7% of patients, half of whom had GERD. Among the remaining 53.3% patients with a voice disorder and no evidence of LPR, GERD was present in 25%. There was no significant association between the presence of LPR based on RSI (P=1) and GERD or RFS and GERD (P=0.06). Proximal acid reflux was present in only 10% patients with a voice disorder, and there was no significant association of this test with RFS (P=1) or RSI (P=1).

**Conclusions:**

Approximately half of the patients with a voice disorder have LPR, and only a subset of these patients have evidence of GERD. Fiberoptic laryngoscopic findings (RFS) complementing RSI appears to be important in diagnosing possible reflux etiology in voice disorders and can be an indicator for instituting anti-reflux therapy. However, there is no significant association between RSI, RFS, and GERD suggesting that these tests evaluate different features of the disease. Proximal acid reflux is uncommon in patients with voice disorder based on current measurement criteria. Acid exposure time as measured in the proximal probe of a 24-hour dual pH probe may need to be re-evaluated as one of the diagnostic criteria for LPR.

## Introduction

Acid reflux is a common problem seen in 4–10% of patients attending ear, nose, and throat (ENT) outpatient departments. Gastroesophageal reflux disease (GERD) is defined as the retrograde flow of gastric contents into the esophagus or above (1,2).GERD is characterized by gastroesophageal reflux symptoms and/or signs of mucosal injury to the esophagus or upper aerodigestive tract (3). Direct physiologic measurement of acid in the esophagus by 24-hour esophageal pH monitoring is the gold standard for the diagnosis of GERD (4).

Otolaryngological manifestations of laryngopharyngeal acid reflux include a wide range of laryngeal and pharyngeal symptoms such as a change in voice, a burning sensation in the substernal/epigastric region, regurgitation, dysphagia, throat pain, cough, foreign-body sensation in the throat, and frequent throat clearing (5,6). Studies of voice problems and reflux disorders reveal that approximately two-thirds of patients with voice problems have laryngopharyngeal reflux (LPR) (7-9). Diagnosis of LPR is made using the Koufman Reflux Symptom Index (RSI), Reflux Finding Score (RFS) based on findings during fiberoptic nasopharyngolaryngoscopy, and proximal acid exposure percentage time by dual-probe pH monitoring (10–13).

As there is no consensus on which test is best for the diagnosis of LPR, the majority of clinicians depend on clinical symptoms and response to empirical therapy with proton pump inhibitors (PPI) to make the diagnosis (14). There is, therefore, a need for further studies to help plan a diagnostic strategy for this common condition. The aims of this study were (a) to determine the frequency of LPR in patients with voice disorders and (b) to determine the association between Koufman RSI, RFS, GERD and proximal acid reflux in these patients.

## Materials and Methods

This was a prospective, descriptive, cross-sectional study. The subjects were patients aged 18 years or above who attended the ENT outpatient clinics of our hospital (tertiary care center) with a history of change in the voice lasting more than 3 weeks. Patients with laryngeal papillomatosis, carcinoma larynx, vocal cord palsy, hypothyroidism, neurological deficits causing a change in the voice, chronic pulmonary disease, asthma, heart disease, scleroderma, pregnant women, or those who had recently received PPIs, H_2_ receptor antagonists, calcium channel blockers, anti-dopaminergic drugs such as domperidone, α- or β-blockers, and those allergic to any anesthetic agent were excluded from the study. Patient details including age, gender, profession, level of voice use, history of voice abuse, addiction, diet, and drug use were obtained (15–17). Patients then underwent nasopharyngolaryngoscopy, auditory perceptual voice evaluation by a speech therapist (18), and 24-hour dual-probe esophageal pH monitoring (19).Tests used to evaluate LPR were: 1) Koufman RSI: calculated using a questionnaire evaluating the severity of symptoms of laryngopharyngeal reflux. An RSI score of ≥13 suggests laryngopharyngeal reflux (10); 2) RFS: calculated from findings suggestive of laryngopharyngeal reflux obtained during flexible nasopharyngolaryngoscopy. An RFS score of ≥7 suggests laryngopharyngeal reflux (11); 3) Dual-probe pH monitoring (Sandhill Scientific Inc., Denver, CO, USA): used to measure 24-hour pH of the proximal and distal esophagus (13). The position of the pH probe was confirmed using videofluoroscopy. A DeMeester Johnson score (DJS) of ≥14.7 was diagnostic of GERD (4). An acid exposure time in the proximal esophagus (proximal probe of pH catheter) of >0.02% suggests LPR (13).

Informed consent was obtained from all patients enrolled in the study. Institutional Review Board approval was secured before undertaking the study.

*Statistical analysis*

Categorical data are presented as frequency with percentages. Continuous data with normal distribution are presented as mean with standard deviation, while non-normally distributed data are presented as median with range. Comparison between categorical variables was performed using Fisher’s exact test. Continuous variables were compared using the Mann Whitney-U test. A p-value of ≤0.05 was considered statistically significant. Statistical analysis was performed using SPSS for Windows v13.

## Results

Thirty patients with voice disorders were included in the study. The mean age of participants was 38.5+10 years and 40% were female. Table 1 shows the baseline characteristics and results of investigations of the study subjects. The median duration of change in voice was 10.5 months (range, 1–120 months). Most patients were level-III or IV voice users. Approximately one-quarter of participants had a history of smoking.

**Fig1:**
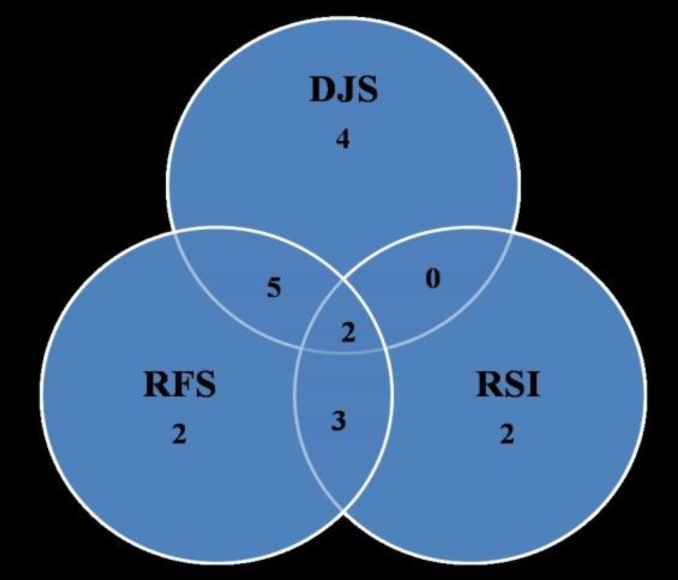
Relationship of positive tests for GERD (DJS) and LPR (RSI and RFS) among patients with voice disorder

LPR was present in 12 patients based on RFS criteria and in seven patients based on RSI criteria (Table 1 and Figure 1). When either of the two criteria were used, a total of 14 (46.7%) patients had LPR, and the remaining patients with voice disorder had no evidence of LPR. Among these 14 patients, only seven (50%) had evidence of GERD based on DJS. In addition, there were four patients with a voice disorder who had GERD on DJS but no features of LPR according to RSI and RFS (Fig.1).

The relationship between the tests for LPR (RFS and RSI) and GERD is shown in Figure 1. The poor overlap suggests a lack of agreement between these tests. The association between the tests was further explored statistically (Tables 2,3). There was a poor association between RSI and GERD (P=1). RFS had a better association with GERD, but it was still short of statistical significance (P=0.06). The two tests for LPR also showed no significant association with each other (P=0.08).

**Table 1:** Baseline characteristics and results of various tests in study subjects (n=30).

**Characteristic**	
Age (mean±SD)	38.5±10 years
Gender (female)	12 (40%)
Duration of symptom (median, range)	10.5 (1–120) months
Level of voice use (I/II/III/IV)	0 (0%) / 6 (20%) / 11 (36.7%) / 13 (43.3%)
History of smoking	7 (23.3%)
Alcohol use	2 (6.7%)
GERD (DJ score)	11 (36.7%)
LPR (RFS)	12 (40%)
LPR (RSI)	7 (23.3%)
Proximal esophageal acid reflux	3 (10%)
	

GERD: Gastroesophageal reflux disease; DJ: DeMeester Johnson; LPR: Laryngopharyngeal reflux; RFS: Reflux Finding Score; RSI: Reflux Symptom Index

**Table 2:** Association between GERD and LPR (RFS or RSI) among patients with voice disorders

		GERD Present	GERD Absent	P-value
RFS	Positive	7	5	0.06
	Negative	4	14	
RSI	Positive	2	5	1.0
		
		
	Negative	9	14	

GERD: Gastroesophageal reflux disease; LPR: Laryngopharyngeal reflux; RFS: Reflux Finding Score; RSI: Reflux Symptom Index

**Table 3:** Association between RFS and RSI

	**RSI positive**	**RSI negative**	**P (Fisher’s exact test)**
RFS Positive	5 (41.7%)	7 (58.3%)	0.08
RFS Negative	2 (11.1%)	16 (88.9%)	

RFS: Reflux Finding Score; RSI: Reflux Symptom Index

The frequency of proximal acid reflux using the 24-hour dual-probe esophageal pH study was very low in patients with voice disorders (10%) based on the proposed criteria of >0.02% acid exposure time in the proximal esophagus. Out of these three patients, one had abnormal RFS, none had abnormal RSI, and one had abnormal GERD based on DJS. The presence of proximal esophageal acid reflux had no significant association with LPR tests including RSI (P=1) or RFS (P=1).

## Discussion

LPR, a supra-esophageal manifestation of GERD, is believed to be caused by the retrograde flow of the contents of the stomach into the laryngopharynx. A large study from Scotland showed significant correlation between GERD and LPR supporting the role of acid reflux in LPR (19). It is difficult to compare the results of different studies on LPR, GERD, and voice disorders because the criteria for diagnosis of the above conditions vary between studies (7,20–22).

Data on the population prevalence of LPR is scarce. A study from Greece estimated it to be around 18.8% based on RSI criteria (23), while a second Greek study reported the prevalence of LPR to be 8.5% (24). In the UK, Kamani et al. reported the community prevalence of LPR to be 30% based on a lower RSI score of >10 (25). The mean age of the patients with LPR in our study (39 years) was lower than that reported in other studies. De Bortoli et al. from Italy reported a mean age of patients with LPR of 51.5±12.7 years, while Spantidias et al. from Greece found the mean age of their patients to be 46.86±14.54 years (range, 21–23). The younger age of our patients may be one of the reasons for the lower frequency of GERD found in our study.

We noted evidence of LPR (positive RSI or RFS) in approximately half (47%) of the study patients with voice disorders. Seven of the 14 patients with LPR had evidence of GERD in the pH study. Overall, one-quarter of our patients with voice disorders had both LPR and GERD, suggesting that acid reflux was the cause of LPR in those patients. We also had four patients with voice disorder and GERD but no evidence of LPR. Studies evaluating the frequency of LPR and GERD in patients with voice disorders show wide-ranging results. In a study from the USA, Koufman et al. evaluated 113 patients with laryngeal and voice disorders and found LPR in 69%; among patients with LPR, 73% had GERD (7). Another study from the USA in patients with chronic hoarseness of voice found GERD in 78.8% subjects (27). An Asian study from the Middle East detected GERD in 80% of patients with a voice disorder, despite using only endoscopy without pH measurement (20). In a study on patients with hoarseness and/or globus pharyngeus from Korea, Park et al. noted that 69% showed abnormal laryngoscopic findings, 58.1% showed abnormal endoscopic findings, and 80% showed a therapeutic response to acid suppression therapy (8). In contrast, de Bortoli from Italy reported a very low frequency (12.2%) of GERD on pH studies in patients with LPR (21). Another study from Malaysia detected GERD on a pH study in 25% patients with chronic laryngitis (28). While the frequency of LPR and GERD may vary in different studies, it is interesting to note that approximately 25–50% of patients with voice disorders have no evidence of LPR. In a study of voice disorders, Randhawa et al. observed that 20% had LPR and 67% had an allergy as the cause of the voice disorder (29). However, as the bulk of studies show that LPR is the major cause of voice disorders, it may be prudent to initially treat patients with a PPI and look for other causes if there is no response to therapy.

In this study, we divided the patients into four groups based on level of voice use as graded by Koufman and Blalock, depending on their profession (15). Most patients were low-level voice users (24 patients [80%] were level-III and IV voice users and six patients [20%] were level-II voice users) suggesting that excessive voice use was not a major risk factor for the voice disorder in our patients. Among patients with GERD in our study, only one (9%) occurred in a level-II voice user and 10 (91%) occurred in level-III and IV voice users, suggesting that GERD and not voice abuse was responsible for voice disorders in the majority of our GERD patients.

Our study showed that RFS had the highest yield for diagnosis of LPR (40%) compared with RSI (23%) and esophageal pH (37%). The data on the comparative yields of RSI, RFS, and esophageal pH in diagnosis of LPR varies in different studies. Dymek et al. showed that RFS (cut-off ≥7) had a lower yield in the diagnosis of LPR as compared with RSI (30)**. **Jette et al. and Randhawa et al. observed that RFS was not specific for LPR, as similar laryngoscopic findings were seen in other inflammatory diseases (29,31). Mesallam et al. found good correlation between RFS and RSI (6). Tables 2 and 3 show no association between RSI, RFS, and esophageal pH (GERD) in our study. This is similar to the observations of Vardar et al., who also found no correlation between Reflux Symptom Score, RSI and RFS (26). The absence of association is because the tests evaluate different features of the disease. The above data also suggest that no single diagnostic test is accurate for the diagnosis of LPR. Therefore, the present diagnostic strategy for LPR may be to perform all tests (RSI, RFS, esophageal pH) as well as to evaluate response to empirical therapy with PPI. Further studies are required to develop a better strategy for the diagnosis of LPR.

An interesting observation was the very low frequency of proximal esophageal acid reflux (10%) in our study subjects. One hypothesis to explain the low incidence of proximal acid reflux on dual-probe esophageal pH monitoring (an accepted confirmatory test for LPR) is that the laryngopharyngeal epithelium is more sensitive to reflux-related injury than the esophageal epithelium. Therefore, smaller amounts of acid and fewer episodes of reflux may be capable of causing damage and laryngeal symptoms. This has led to suggestions to reconsider the cut-off value used for diagnosis of proximal acid reflux (32). Further, Hila et al. have shown that a pH study is not very sensitive for the detection of weak acidic reflux (33). Another potential mechanism for supra-esophageal manifestations of GERD is vasovagal reflex triggered by acidification of the distal esophagus by micro-aspirations not detected by a proximal and distal probe. The vasovagal reflex induces bronchospasm, cough, foreign-body sensation in the throat, and frequent throat clearing (34). Other reasons for a negative study are the improper positioning of the proximal pH probe and non-acid gastroesophageal reflux.

 The strengths of our study are its prospective nature and the comprehensive evaluation of patients according to protocol. Limitations include the relatively small sample size and variable position of the proximal probe in the pharynx due to the fixed length between the distal and proximal probes and the variable length of the esophagus in different patients.

## Conclusion

The frequency of LPR evaluated by RSI and RFS is approximately 47% in patients with voice disorders. This suggests that causes other than gastroesophageal reflux, such as allergy, need to be evaluated in approximately 25–50% of patients with voice disorders. There is no significant statistical association between RSI, RFS, and gastroesophageal acid reflux, suggesting that these tests evaluate different features of the disease. The yield from 24-hour dual-probe pH monitoring (acid exposure in the proximal probe of the of >0.02%) for the diagnosis of LPR is low, and changes in diagnostic criteria need to be considered. Further studies are required to help develop a stra
